# 

**Published:** 1863-05

**Authors:** 


### CONTENTS.

ORIGINAL ESSAYS AND COMMUNICATIONS.

Metals and Alloys. By Dr. B. Wood........ 193
The Use of the Mallet, By Dr. W. H. Atkinson........ 199
"An Experiment" Examined. By Dr. B. Wood........ 203
Practical Thoughts. By Dr. Wm. A. Pease........ 209

CORRESPONDENCE.

Treatment of Fangs........ 215

SELECTIONS.

Anaesthetica........ 218
Specialists in Medicine........ 224
Steatomatous Cyst upon the Tongue........ 226
On the Introduction of Specialities into Bellevue Hospital........ 227
Parched Corn Meal........ 229
Treatment of Hare Lip........ 230
"Que va a la Chasse perd sa place"........ 231
Sensible Advice........ 232

EDITORIAL.

"Practical Thoughts"........ 233
Bibliographical........ 236

